# Radiological findings based comparison of functional status in patients who have post-covid lung injury or idiopathic pulmonary fibrosis

**DOI:** 10.1186/s12890-023-02527-z

**Published:** 2023-06-30

**Authors:** Deniz Kızılırmak, Seçil Sarı, Fatma Can, Yavuz Havlucu

**Affiliations:** 1 Faculty of Medicine, Chest Diseases Department, Manisa Celal Bayar University, Manisa, Turkey; 2 Hafsa Sultan Hospital, Respiratory Therapist, Manisa Celal Bayar University, Manisa, Turkey; 3 Faculty of Medicine, Department of Radiodiagnostics, Manisa Celal Bayar University, Manisa, Turkey

**Keywords:** COVID-19, Lung injury – IPF − 6MWT

## Abstract

**Background:**

Following COVID-19 infection, some patients acquired lung injury and fibrosis. Idiopathic pulmonary fibrosis is characterized by lung fibrosis. Both post-COVID lung injury and idiopathic pulmonary fibrosis cause loss of respiratory function and involvement of the lung parenchyma. We aimed to compare respiratory related functional characteristics and radiological involvement between post-COVID lung injury and idiopathic pulmonary fibrosis.

**Methods:**

A single center, cross-sectional study was applied. Patients with post-COVID lung injury and idiopathic pulmonary fibrosis included in the study. All patients underwent the 6-minute walk test, as well as the Borg and MRC scales. Radiological images were evaluated and scored for lung parenchymal involvement. The impact of post-COVID lung injury and idiopathic pulmonary fibrosis on respiratory functions of were compared. The relationship of functional status and radiological involvement, as well as the effect of potential confounding factors were investigated.

**Results:**

A total of 71 patients were included in the study. Forty-eight (67.6%) of the patients were male and the mean age was 65.4 ± 10.3 years. Patients with post-COVID lung injury had greater 6-minute walk test distance and duration, as well as higher oxygen saturations. The MRC and Borg dyspnea scores were comparable. At radiologic evaluation, ground glass opacity scores were higher in patients with post-COVID lung injury, whereas pulmonary fibrosis scores were higher in patients with idiopathic pulmonary fibrosis. However, the total severity scores were similar. While pulmonary fibrosis score was found to have a negative correlation with 6-minute walk test distance, test duration, and pre- and post-test oxygen saturation levels, there was a positive correlation with oxygen saturation recovery time and MRC score. There was no relationship between ground glass opacity and the functional parameters.

**Conclusions:**

Despite having equal degrees of radiological involvement and dyspnea symptom severity, PCLI patients exhibited higher levels of functional status. This might be due to different pathophysiological mechanisms and radiological involvement patterns of both diseases.

## Background

More than 532 million people worldwide were infected with COVID-19, caused by the SARS-COV-2 virus. It was seen that approximately 15% of people with COVID-19 had a severe course of the disease causing acute respiratory failure and/or multi-organ failure in 5% of patients [1]. Although the mortality rate due to COVID-19 was higher compared to other respiratory viral infections, most patients infected with SARS-COV-2 recovered after the acute phase. Due to the lack of alveolar re-epithelialization, activation of fibroblasts, collagen, and other extracellular matrix deposition after COVID-19 infection, long-term pulmonary lung injury and fibrosis developed in some patients [2]. The period from 4 to 12 weeks is called as “ongoing symptomatic COVID-19” and the period 12 weeks after the infection is considered as the “post-COVID period” [3]. In the long term, a significant number of patients with COVID-19 appear to suffer from anxiety, depression, fatigue, loss of cognitive function and post-COVID lung injury (PCLI) with pulmonary parenchymal abnormalities, respiratory disfunction and reduced physical capacity.

Idiopathic pulmonary fibrosis (IPF) is characterized by fibroblast and myofibroblast accumulation and fibrosis in the alveolar tissue. IPF causes restriction due to fibrosis and loss of respiratory function. Dyspnea at rest and increasing with effort, oxygen desaturation with exercise, effort limitation and decrease in functional capacity are common in IPF patients [2].

In this study, we aimed to compare patients with PCLI and those with IPF who have not had COVID-19 pneumonia before in terms of respiratory-related functional parameters.

## Methods

A single center, cross-sectional study was applied. PCLI and IPF patients followed in the Chest Diseases Outpatient Clinic in a university hospital included in the study. Inclusion criteria were determined as being older than 18 years of age, having had microbiologically proven COVID-19 infection, having passed at least 12 weeks after COVID-19 infection and giving consent to participate in the study for PCLI patients. IPF patients who were in the same age range and accepted to be included in the study were included in the study as the active control group. IPF patients were selected from follow-up patients diagnosed by a multidisciplinary evaluation of those with clinically compatible and usual radiological findings of interstitial pneumonia. Patients with microbiologically unproven COVID-19 infection, with lack of data, pregnant women, those without cooperation and mental disability, with active malignancies that have not entered the remission period, who received continuous oxygen therapy before COVID-19 infection, patients with systemic rheumatological, endocrinological and hematological diseases (except diabetes mellitus), those with chronic lung disease or congestive heart failure prior to COVID-19 infection, with a history of lung surgery, with orthopedic problems that prevent walking test, patients without computed tomography results, with a history of pulmonary rehabilitation, Post-COVID Lung Injury patients who received antifibrotic therapy among COVID-19 infection and IPF patients with a history of COVID-19 pneumonia were excluded from the study.

**Fig. 1:**
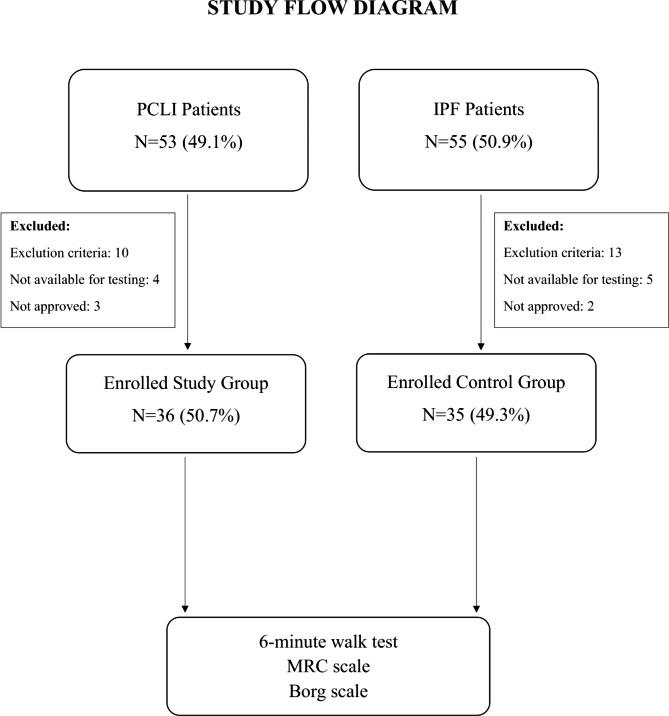
Study flow diagram. PCLI: Post-COVID lung injury, IPF: Idiopathic pulmonary fibrosis

Ethics committee approval was obtained from Manisa Celal Bayar University, Medicine Faculty, Clinical Researches Ethical Committee (Decision date: 02.01.2023, Decision number: 376). Among the patients with PCLI in the Post-COVID period and patients with IPF followed in the Chest Diseases Outpatient Clinic, those meeting the inclusion criteria were informed about the study. After the “Informed Consent Form” was signed by the patients who gave consent to participate in the study and did not meet any exclusion criteria, the “Demographic Data and Registration Form” was filled. Then, 6-minute walk test was applied to the patients and the Medical Research Council (MRC) and Borg dyspnea scales were applied at the same visit. Demographic and medical data were administered by the field investigator. 6-minute walk test and scales were applied by the respiratory therapist (Fig. 1).

Before application of 6-minute walk test, the patient was seated in a chair and rested for 15 minutes. The 6-minute walk test was performed as the patient walked for 6 minutes in a corridor of a certain length, where the start and end points were marked [4]. Blood pressure, heart rate, oxygen saturation, and Borg dyspnea index scores were recorded before and after the 6-minute walk test. Also, the times it took for oxygen saturation and heart rate to return to the resting values were calculated. Borg is a scale on which the patient scores current dyspnea on a scale of 0–10. “0” indicates no shortness of breath, and “10” indicates maximum degree of dyspnea [5]. In the MRC dyspnea scale, the MRC grades mean “1: I get short of breath only during strenuous exercise, 2: I get short of breath only when I walk fast on the flat road or when I go up a slight hill, 3: I walk slower than my peers on the flat road or stop and rest from time to time due to my shortness of breath, 4: I get short of breath after walking 100 meters or for a few minutes on the straight road and I stop, 5: I can’t leave the house because of shortness of breath or I have shortness of breath when I dress and undress.”, respectively [6].

Radiological images of the patients, taken in the post-COVID period or during IPF follow-up, were evaluated by a radiologist without knowing the diagnosis and clinical information of the patients and were scored in terms of lung parenchymal involvement. Septal thickening, traction bronchiectasis and ground glass areas including honeycomb and presence of pulmonary fibrosis were evaluated for the severity of involvement. These were scored using Franquet’s computed tomography scoring system. Separate scores were made for ground glass opacity and pulmonary fibrosis. Each lobe is scored separately between 0 and 3 points. The lingula was evaluated as a separate lobe. Ground-glass opacity and pulmonary fibrosis scores were summed for the total severity score. The total severity score was used to determine the total degree of involvement [7].

Study participant numbers were given to the patients and their personal data were kept confidential. Medical data and test results of the patients were analyzed with the “IBM SPSS Statistics for Macintosh, Version 26.0” program without using personal data [8]. Sample size calculation for comparison of means was done with G*Power 3.1.9.7 program, with type 1 error (alpha) of 0.05, type 2 error (beta) of 0.20 and effect size of 0.73 as 31 per group [9]. Continuous variables with and without normal distribution were analyzed by Independent Samples t-test and Mann Whitney U test, respectively. Chi-square test was used for categorical variables. Pearson correlation test was used for correlation analysis. Multiple linear regression analysis was used to adjust the association between PCLI and functional status variables for confounders. The power of the study was calculated as 86% in the post-hoc analysis.

The impact of PCLI and IPF on dyspnea symptoms and respiratory functions of patients and the factors affecting the results were compared with MRC and Borg scales and 6-minute walk test results. The relationship of functional status and radiological involvement between two groups and effect of possible confounding factors were investigated.

## Results

A total of 71 patients were included in the study. Forty-eight (67.6%) of the patients were male and the mean age was 65.4 ± 10.3 years. Thirty-seven (52.1%) patients were smokers and had a median (25–75%) exposure of 30 (25–60) pack-years. Of the patients, 36 (50.7%) had hypertension, 10 (14.1%) cardiovascular disease and 22 (31.0%) diabetes mellitus. Twenty-three (63.9%) of the PCLI patients had a history of hospitalization and 7 (19.4%) had intensive care unit admission history. Thirteen (37.1) of IPF patients were receiving antifibrotic therapy. Six patients were using Pirfenidone and 7 patients were using Nintedanib at the time the study was carried out.

Age of PCLI patients was lower (63 ± 12 vs. 68 ± 7 years; p = 0.02) and IPF patients had higher percentage of hypertension (36.1% vs. 65.7%; p = 0.02). Other characteristic features were found to be similar (Table 1).

**Table 1:** Characteristics of PCLI and IPF patients

	PCLI (N = 36)	IPF (N = 35)	p value
**Age, years, mean ± SD**	63 ± 12	68 ± 7	**0.02**
**Gender**			
Male, n (%)	25 (69.4)	23 (65.7%)	0.80
**Smoking status**			
Smoker, n (%)	21 (58.3)	16 (45.7)	0.35
Average pack-year, median (25-75%)	30 (25–60)	40 (30–50)	0.62
**Body mass index**, kg/m^2^, mean ± SD	27.4 ± 4.5	27.2 ± 4.1	0.86
**Concomitant diseases n (%)**			
Hypertension	13 (36.1)	23 (65.7)	**0.02**
Cardiovascular diseases	3 (8.3)	7 (20.0)	0.19
Diabetes mellitus	12 (33.3)	10 (28.6)	0.80

PCLI: Post-COVID lung injury, IPF: Idiopathic pulmonary fibrosis, ICU: Intensive care unit

6-minute walk test distance and duration of PCLI patients were significantly higher. PCLI patients walked an average of 371.1 ± 127.3 m, while IPF patients walked 221.7 ± 154.9 m (p < 0.001). The test completion time was 332.2 ± 81.1 s in PCLI patients and 202.9 ± 135.1 s in IPF patients. IPF patients had to terminate the test sooner (p < 0.001). Oxygen saturation values before and after the 6-minute walk test were 95.7 ± 2.6% and 91.5%±4.6% in PCLI patients, 91.5%±4.6% and 86.7 ± 4.7% in IPF patients, respectively. Both values were significantly higher in PCLI patients (p = 0.001, p < 0.001, respectively). These differences were also statistically significant when adjusted for age, gender, smoking status and body mass index by linear regression analysis.

Heart rate levels before and after the 6-minute walk test, oxygen saturation and heart rate recovery times and the pre-test Borg dyspnea index grades were similar in both groups. Although the MRC and Borg scores after the 6-minute walk test were lower in the PCLI group in univariate analysis, both groups were statistically similar when adjusted for age, gender, smoking status and body mass index.

The ground glass opacity score was 7.9 ± 5.7 in PCLI patients and 2.3 ± 2.0 in IPF patients, which was significantly higher in PCLI patients (p < 0.001). In contrast, the pulmonary fibrosis score was higher in IPF patients with 6.5 ± 4.1 versus 10.8 ± 2.9 (p < 0.001). The total severity score of lung involvement was found to be similar in both groups (p = 0.17) (Table 2).

**Table 2:** Functional parameters and radiological involvement of PCLI and IPF patients

Outcomes	PCLI	IPF	PCLI-IPF crude difference (95%CI)	PCLI-IPF adjusted difference (95%CI)*
**6-minute walk test**				
Walking distance, meters, mean ± SD	371.1 ± 127.3	221.7 ± 154.9	**149.4 (82.3 to 216.5)**	**109.3 (42.6 to 176.0)**
Duration, seconds, mean ± SD	332.2 ± 81.1	202.9 ± 135.1	**129.3 (76.7 to 181.9)**	**112.9 (56.9 to 169.0)**
Pre-test oxygen saturation, %, mean ± SD	95.7 ± 2.6	93.2 ± 3.7	**2.5 (1.0 to 4.0)**	**2.4 (0.8 to 4.1)**
Post-test oxygen saturation, %, mean ± SD	91.5 ± 4.6	86.7 ± 4.7	**4.8 (2.7 to 7.0)**	**4.1 (1.8 to 6.5)**
Oxygen saturation recovery, seconds, median (25-75%)	8 (1–31)	23 (8–46)	-11.6 (-23.5 to 0.2)	-7.6 (-20.2 to 4.9)
Pre-test heart rate, per minutes, mean ± SD	105.3 ± 16.5	102.3 ± 18.2	3.0 (-5.2 to 11.2)	-0.4 (-8.8 to 8.0)
Post-test heart rate, per minutes, mean ± SD	118.8 ± 19.2	116.0 ± 14.6	2.8 (-5.3 to 10.9)	-0.7 (-8.7 to 7.3)
Heart rate recovery, seconds, median (25-75%)	5 (0–15)	8 (0–14)	-1.4 (-5.1 to 2.4)	-0.8 (-4.7 to 3.1)
Pre-test Borg score, median (25-75%)	2 (1–3)	3 (2–3)	-0.3 (-0.9 to 0.3)	-0.2 (-0.9 to 0.4)
Post-test Borg score, median (25-75%)	4 (2–5)	5 (3–7)	**-1.1 (-2.2 to -0.1)**	-0.9 (-2.0 to 0.2)
**MRC score, mean ± SD**	3.8 ± 1.2	4.3 ± 1.0	**-0.5 (-1.1 to -0.2)**	-0.2 (-0.7 to 0.3)
**Radiological findings**				
Ground glass opacity score, mean ± SD	7.9 ± 5.7	2.3 ± 2.0	**5.7 (3.6 to 7.7)**	**5.3 (3.2 to 7.4)**
Pulmonary fibrosis score, mean ± SD	6.5 ± 4.1	10.8 ± 2.9	**-4.3 (-6.0 to -2.6)**	**-3.8 (-5.5 to -2.1)**
Total severity score, mean ± SD	14.4 ± 6.0	12.8 ± 3.7	1.7 (-0.7 to 4.0)	1.7 (-0.9 to 4.3)

PCLI: Post-COVID lung injury, IPF: Idiopathic pulmonary fibrosis, SD: Standard deviation, MRC: Medical research council

*: Adjusted for age, gender, smoking status and body mass index

Although the total lung involvement severity score was similar in both groups, the functional capacity scores was lower in IPF patients. Therefore, the effect of lung involvement pattern on functional parameters was evaluated. While a negative correlation was found between fibrosis score to 6-minute walk test distance, test duration and pre-test and post-test oxygen saturation levels; there was a positive correlation to oxygen saturation recovery time and MRC score. There was no correlation between the ground glass opacity score and functional parameters (Table 3).

**Table 3:** Correlation of lung involvement scores with functional parameters

Outcomes	Ground glass opacity score r, p	Pulmonary fibrosis score r, p	Total severity score r, p
**6-minute walk test**			
Walking distance, meters	0.04, 0.75	**-0.37, 0.002**	**-0.24, 0.04**
Duration, seconds	0.13, 0.29	**-0.34, 0.004**	-0.17, 0.15
Pre-test oxygen saturation, %	0.09, 0.48	**-0.31, 0.01**	-0.18, 0.14
Post-test oxygen saturation, %	0.14, 0.25	**-0.45, < 0.001**	-0.22, 0.07
Oxygen saturation recovery, seconds	0.10, 0.40	**0.30, 0.01**	**0.32, 0.007**
Pre-test heart rate, per minutes	0.21, 0.07	0.08, 0.51	**0.33, 0.005**
Post-test heart rate, per minutes	0.21, 0.08	-0.15, 0.21	0.14, 0.23
Heart rate recovery, seconds	0.03, 0.81	-0.17, 0.16	-0.15, 0.22
Pre-test Borg score	0.03, 0.80	0.08, 0.49	0.11, 0.35
Post-test Borg score	-0.07, 0.53	0.22, 0.06	0.09, 0.46
**MRC score**	-0.12, 0.30	**0.40, < 0.001**	0.21, 0.09

MRC: Medical research council

## Discussion

This study shows that even though the severity of dyspnea symptoms and total lung involvement of the patients were identical, the 6-minute walk test distance, duration to complete the test, and oxygen saturation levels at rest and after the 6-minute walk test were all higher in PCLI patients. It is an interesting finding that these differences persist even when adjusting to age, gender, smoking status and body mass index in patients with IPF who are older and for whom smoking is a significant risk factor.

There are some studies showing that functional capacity is affected in patients in the post-COVID period [10]. However, there is no study in the literature that compares PCLI and IPF. In the study of Gonzales et al., the median 6-minute walk test distance was 400 m and radiological abnormalities were detected in 70.2% of the patients in the evaluation 3 months after discharge in patients who developed acute respiratory distress syndrome due to COVID-19 [11]. In our study, functional capacity decreased with a mean distance of 371.1 ± 127.3 m similarly. It was shown that radiological damage and functional loss continued in the later stages of the post-COVID period. Respiratory function, functional capacity, quality of life and fatigue levels of the individuals with severe COVID-19 infection were found to be impaired at 6 months after ICU discharge [12]. It was shown that radiological involvement persisted in 56.7% and pulmonary diffusion restriction persisted in 26.1% of the patients in the 1st year after COVID-19 infection [13]. In another study, 81% of severely ill patients and 37% of moderately ill patients showed residual abnormalities 12 months after COVID-19 infection [14]. These results support that our study, which shows functional deterioration in the early period of the post-COVID period, sheds light on the future results.

The fact that the ground glass opacity areas in PCLI patients and pulmonary fibrosis areas in IPF patients are more common in the radiological evaluation may be significant in terms of explaining this situation. As a matter of fact, in the correlation analysis, ground glass areas were not found to be associated with dyspnea symptoms and functional parameters, but the prevalence of pulmonary fibrosis was found to be negatively correlated with respiratory functions.

In studies performed on IPF patients, pulmonary function tests showed an inverse correlation with reticular pattern and honeycomb areas, but no correlation with ground glass areas [15, 16]. Another study investigating the radiological involvement patterns and the results of the 6-minute walk test and pulmonary function test in IPF patients found the extent of reticular opacity to be associated with forced vital capacity (FVC), but the extent of ground glass areas was not found to be related. However, the extent of both reticular opacity areas and ground glass opacities were found to be inversely correlated with the 6-minute walking test distance [17]. This may be since the study included only IPF patients with the usual pattern of interstitial pneumonia. In our study, which included both ground glass opacity and pulmonary fibrosis patient groups, only pulmonary fibrosis was found to be correlated with respiratory functions.

In genomic studies, some up-regulated homologous chemokines such as CXCL9, CXCL10 and CCL5, which are involved in the regulation of immune cell migration and activation in IPF, were also detected in COVID-19 patients. Furthermore, increased expression of some membrane G proteins with intracellular calcium-related downstream signaling functions showed a similar functional association to those in IPF. This situation could predict a similar clinical picture for the two diseases. However, differences were found in the number of genes and isoform types, and it was thought that they would cause different types of fibrogenic response [18]. As a matter of fact, it suggests that a higher proportion of patients with COVID-19 will not eventually develop a fibroproliferative process similar to IPF, and that the parenchymal involvement observed in COVID-19 patients will fit other fibrotic pneumopathies with more favorable prognoses such as organizing pneumonia or proliferative diffuse alveolar damage [19]. In our study, PCLI patients differed significantly from IPF patients in terms of both parenchymal involvement pattern and effects on functional parameters, which supports the expectations of genomic and physiopathological studies.

Performing pulmonary function tests and measuring the diffusion capacity in particular would be very useful in terms of an objective demonstration of pulmonary functions. Since the COVID-19 pandemic, pulmonary function tests could not be performed due to the risk of viral transmission. The main limitations of the study are that the patients were evaluated at one visit, the functional status of the patients before the COVID-19 infection could not evaluated and it did not give an idea about the future processes of the diseases. The prominent aspects of the study can be listed as the exclusion of confounders that would affect the functional status, the fact that the patients had similar characteristic levels, the functional and radiological parameters of the patients were evaluated with objective parameters and the high impact power of the study. Cohort studies comparing the future status of PCLI and IPF patients may contribute to the clarification of this issue.

## Conclusions

In conclusion, while the severity of dyspnea symptoms and radiological involvement were comparable, PCLI patients had higher functional status parameters. The severity of radiological involvement and the patient’s experience of dyspnea had no effect on the greater functional loss in IPF patients. It is only related to the severity of the pulmonary fibrosis.

This might be due to the different pathophysiological mechanisms and radiological involvement patterns of both diseases. The main limitations were the unknown functional status of the patients prior to COVID-19 infection and the lack of longitudinal follow-up. It is recommended to conduct prospective studies investigating disease mechanisms and progression.

## Data Availability

The datasets generated during and/or analysed during the current study are not publicly available but are available from the corresponding author on reasonable request.
